# Characteristics of sports and recreation-related emergency department visits among school-age children and youth in North Carolina, 2010–2014

**DOI:** 10.1186/s40621-018-0152-0

**Published:** 2018-05-15

**Authors:** Katherine J. Harmon, Scott K. Proescholdbell, Johna Register-Mihalik, David B. Richardson, Anna E. Waller, Stephen W. Marshall

**Affiliations:** 1 Department of Epidemiology, University of North Carolina at Chapel Hill, 2101 McGavran-Greenberg Hall, CB #7435, Chapel Hill, NC 27599-7435 USA; 2 Injury and Violence Prevention Branch, Chronic Disease and Injury Section, NC Division of Public Health, NC Department of Health and Human Services, 5505 Six Forks Road, Raleigh, NC 27609 USA; 3 Department of Exercise and Sport Science, University of North Carolina at Chapel Hill, 125 Fetzer Hall, CB# 8700, Chapel Hill, NC 27599-8700 USA; 4 Matthew Gfeller Center, University of North Carolina at Chapel Hill, 2207 Stallings-Evans Sports Medicine Complex, CB# 8700, Chapel Hill, NC 27599-8700 USA; 5 Department of Epidemiology, University of North Carolina at Chapel Hill, 2102B McGavran-Greenberg Hall, CB# 7435, Chapel Hill, NC 27599-7435 USA; 6 Department of Emergency Medicine, University of North Carolina at Chapel Hill, Physicians Office Building, 170 Manning Dr., CB# 7594, Chapel Hill, NC 27599-7594 USA; 7 Carolina Center for Health Informatics, University of North Carolina at Chapel Hill, Floor 1, 100 Market Street, CB #7597, Chapel Hill, NC 27516 USA; 8 Injury Prevention Research Center, University of North Carolina at Chapel Hill, CVS Plaza, Suite 500, 137 East Franklin Street, CB# 7505, Chapel Hill, NC 27599-7505 USA

**Keywords:** Injury surveillance, Youth sports, Epidemiology, Emergency department, Traumatic brain injury

## Abstract

**Background:**

Sports and recreational activities are an important cause of injury among children and youth, with sports-related traumatic brain injuries (TBIs) being of particular concern given the developing brain. This paper reports the characteristics of sport and recreation-related (SR) emergency department (ED) visits among school-age children and youth in a statewide population.

**Methods:**

This study included all injury-related visits made to all North Carolina 24/7 acute-care civilian hospital-affiliated EDs by school-age youth, 5–18 years of age, during 2010–2014 (*N* = 918,662). Population estimates were based on US decennial census data. Poisson regression methods were used to estimate incidence rates and rate ratios.

**Results:**

During the five-year period, there were 767,075 unintentional injury-related ED visits among school-age youth, of which 213,518 (27.8%) were identified as SR injuries. The average annual absolute number and incidence rate (IR) of SR ED visits among school-age youth was 42,704 and 2374.5 ED visits per 100,000 person-years (95% confidence interval [CI], 2364.4–2384.6), respectively. In comparison to other unintentional injuries among school-age youth, SR ED visits were more likely to be diagnosed with an injury to the upper extremity (Injury Proportion Ratio [IPR] = 1.28; 95% CI, 1.27–1.29), the lower extremity (IPR = 1.14; 95% CI, 1.13–1.15), and a TBI or other head/neck/facial injury (IPR = 1.12; 95% CI, 1.11–1.13).

Among ED visits made by school-age youth, the leading cause of SR injury was sports/athletics played as a group or team. The leading cause of team sports/athletics injury was American tackle football among boys and soccer among girls. The proportion of ED visits diagnosed with a TBI varied by age and sex, with 15–18 year-olds and boys having the highest population-based rates.

**Conclusions:**

Sports and recreational activities are an important component of a healthy lifestyle, but they are also a major source of injury morbidity among school-age youth. Physical activity interventions should take into account sex and age differences in SR injury risk.

## Background

Physical activity, along with a healthy diet, is one of the most commonly suggested solutions to the childhood overweight and obesity epidemic (Ebbeling et al. 2002; Goran et al. 1999). Despite the benefits of physical activity and organized sports participation, there are inherent risks to engaging in physical activity, in particular, risk of injury (Marshall and Guskiewicz 2003). Children and youth bear a disproportionate burden of sports and recreation-related (SR) injuries, with an estimated two-thirds of all medically attended SR injuries occurring among 5–24 year-olds (Centers for Disease Control and Prevention 2002; Conn et al. 2003). As compared to other types of injury, youth are more likely to have a diagnosis of a strain/sprain, fracture, superficial wound/contusion, and traumatic brain injury (TBI), including concussion (Burt and Overpeck 2001). TBIs are of particular concern because of the potential for possible long-term adverse health outcomes (Andruszkow et al. 2014; Babikian and Asarnow 2009; Sariaslan 2016; Taylor et al. 2002).

Most prior studies of SR injuries have used survey or sampling methods to estimate incidence. In addition, many of these publications are greater than 10 years old and have focused on high school and college athletes. This is one of the first studies to use a broad definition to describe the characteristics and incidence of sports and recreation-related injury and TBI among school-age youth in a well-defined population, only.

## Methods

This population-based descriptive epidemiologic study examined the incidence, circumstances, and characteristics of SR injuries in children and youth. All NC ED visits for SR injury made by children 5–18 years of age during the period January 1, 2010 – December 31, 2014 were included. A broad and inclusive definition of SR injury was utilized, based on a public health model that underscores the importance of children developing lifetime patterns of healthy public activity within an environment of effective public health interventions designed to minimize injury risk (Marshall and Guskiewicz 2003).

### Injury ascertainment

The ED visit data were obtained from the North Carolina Disease Event Tracking and Epidemiologic Collection Tool (NC DETECT) for the period January 1, 2010 – December 31, 2014. The Department of Emergency Medicine at the University of North Carolina at Chapel Hill and the NC Division of Public Health operate NC DETECT for the purpose of timely syndromic public health surveillance as mandated under state law since 2005 (Carolina Center for Health Informatics 2017; North Carolina State Government 2004). As of December 31, 2014, NC DETECT collected ED visit data from 123 acute-care, hospital-affiliated, civilian EDs in the state, representing an estimated 99% of total NC ED visits (Carolina Center for Health Informatics 2017; Harmon et al. 2012). During the five-year period of study, NC DETECT collected over 23 million ED visits, with an average of 4.7 million ED visits per year (Carolina Center for Health Informatics 2017).

An ED visits was defined as injury-related if it received an ICD-9-CM External Cause of Injury Code (E-code) indicating a valid mechanism of injury, and unintentional-injury-related if it contained an E-code indicating an injury of an unintentional intent (i.e. an injury not inflicted on purpose; “accidental”) in the range of E800-E869 or E880-E929 (National Center for Injury Prevention and Control 2007; National Center for Injury Prevention and Control 2014). The NC Division of Public Health and the University of North Carolina at Chapel Hill approved this study.

### Case definition of a sports and recreation-related (SR) injury

The E-code-based case definition of SR injury was designed specifically for this study. Table 2 contains a list and brief description of the E-codes included in the definition of SR injury. For certain categories of sports and recreational activities, E-codes were grouped together due to the relatedness of the E-codes and/or due to small numbers of ED visit totals.

NC DETECT captures up to five E-codes for each patient visit. When multiple E-codes met the case definition (Table 2), the ED visit was classified according to the most specific E-code. For example, if an individual patient visit contained the following two E-codes: E849.4 (place for recreation and sport) and E007.0 (American tackle football), then ED visit was classified as being due to American tackle football. If the patient visit contained two E-codes of similar specificity, the visit was classified according to the first listed E-code. For example, if an ED visit contained the following two E-codes: E001.0 (walking, marching and hiking) and E001.1 (running), the ED visit would be classified as being due to walking, marching, and hiking. Table 1 contains the order in which E-codes were assigned, with “1” (e.g. E006.2 “Activities involving golf”) referring to the highest level of specificity and “7” referring to the lowest level of specificity (e.g. E849.4 “Accidents occurring in place for recreation and sport”). Over two-thirds of ED visits contained only one E-code for a SR (68.0%), with 26.0% and 6.0% of ED visits contained two and three E-codes for a SR injury, respectively.

**Table 1:** Selected characteristics of unintentional injury-related ED visits among school-age children: NC, 2010–2014

Characteristics	ED visits due to sports and recreation-related injuries	Total number of unintentional injuries
	(*N* = 213,518)	(*N* = 767,075)
Age, No. (%)		
5–9 years	49,491 (23.2)	232,316 (30.3)
10–14 years	94,875 (44.4)	275,738 (35.9)
15–18 years	69,142 (32.4)	259,021 (33.8)
Sex, No. (%)		
Male	145,005 (67.9)	443,797 (57.9)
Female	68,500 (32.1)	323,228 (42.1)
Missing	13	50
Disposition, No. (%)		
Discharged	202,313 (96.8)	722,624 (96.7)
Admitted^a^	5234 (2.5)	16,871 (2.3)
Died	33 (0.0)	183 (0.0)
Other^b^	1419 (0.7)	7916 (1.1)
Missing	4519	19,481
County urban/rural designations, No. (%)		
Urban	136,871 (64.2)	487,227 (63.6)
Mostly rural	62,117 (29.1)	232,223 (30.3)
Completely rural	4191 (2.0)	14,929 (1.9)
Out-of-state	10,094 (4.7)	31,782 (4.1)
Missing	245	914
Mode of transport, No. (%)		
Walk-in	166,709 (88.2)	681,567 (85.2)
Ambulance^c^	12,784 (6.8)	80,712 (10.1)
Other^d^	9517 (5.0)	37,935 (4.7)
Missing	24,508	118,448
Expected source of payment, No. (%)		
Medicaid	89,376 (42.7)	354,035 (47.2)
Insurance company	80,573 (38.5)	240,262 (32.1)
Self-pay	17,212 (8.2)	73,835 (9.9)
Other^e^	21,913 (10.5)	81,461 (10.9)
Missing	4444	31,348
Month, No. (%)		
Dec. - Feb.	37,622 (17.6)	148,609 (19.4)
March–May	61,015 (28.6)	212,405 (27.7)
June - Aug.	48,733 (22.8)	197,943 (25.8)
Sept. - Nov.	66,148 (31.0)	208,118 (27.1)
Hour of day, No. (%)		
12:00–5:59 AM	8480 (4.0)	42,083 (5.5)
6:00–11:59 AM	28,400 (13.3)	115,875 (15.1)
12:00–5:59 PM	73,124 (34.2)	266,935 (34.8)
6:00–11:59 PM	103,514 (48.5)	342,182 (44.6)

*Abbreviations*: *ED* emergency department, *no.* number, *Dec*. December, *Feb.* February, *Aug.* August, *Sept.* September, *Nov.* November

^a^The category of admitted includes transfers to other facilities

^b^The category other disposition contains left against medical advice, left without being seen, observation unit, and other disposition

^c^The category ambulance contains ground, helicopter, fixed wing, other type of ambulance, and unspecified type of ambulance

^d^The category other mode of transport contains other mode of transport

^e^The category other expected source of payment contains Medicare, no charge, other form of government payment, workers’ compensation, and other expected source of payment

### Case definition of a traumatic brain injury (TBI)

This study used the Centers for Disease Control and Prevention (CDC) TBI case definition to identify ED visits due to TBIs. The CDC TBI definition comprises ICD-9-CM codes 800.00–801.99 (fracture of the vault or base of the skull); 803.00–804.99 (other or multiple fractures of the skull); 850.0–850.9 (concussion); 851.00–854.99 (intracranial injury); 950.0–950.9 (optic chiasm, optic pathways, or visual cortex); and 959.01 (head injury, unspecified) (Marr and Coronado 2004). The following code was excluded from the definition: 995.55 “shaken infant syndrome”. As NC DETECT collects up to eleven ICD-9-CM diagnosis codes per patient visit, the ED visit was classified as being due to a TBI if the visit contained a TBI diagnosis code in any position. The majority of ED visits contained one diagnosis code for TBI (90.8%) while the maximum number of diagnosis codes for TBI was five (0.01%).

### Covariates

Sociodemographic covariates included patient sex, patient age (categorized as 5–9, 10–14, and 15–18 years), and urban-rural classification based on NC county of residence. Patient county of residence was classified according to US Census urban-rural designations (urban, mostly rural, completely rural, and out-of-state) (US Census Bureau 2016).

This study also examined discharge disposition (admitted, died, discharged from the ED, and other disposition), mode of transport (walk-in, ambulance, and other specified mode of transport), expected source of medical payment (Medicaid, insurance company, self-pay, and other specified source of payment), seasonality of visit (December–February, March–May, June–August, and September–November), and time of visit (12:00–5:59 AM, 6:00–11:59 AM, 12:00–5:59 PM, and 6:00–11:59 PM).

Injury diagnoses were categorized using the Barell Injury Diagnosis Matrix according to the nature of injury (e.g. fracture) and location (e.g. upper extremity) using the first listed ICD-9-CM injury diagnosis code (Barell et al. 2002).

### Statistical analysis

This study used descriptive epidemiologic methods such as the Pearson’s chi-square test and Fisher’s exact test (for expected cell counts < 5) to characterize SR ED visits. In addition, injury proportion ratios (IPRs) and 95% confidence intervals (CIs) were calculated to compare differences among Barell Injury Diagnosis Matrix classifications between SR injuries and other unintentional injuries. All 95% CIs not containing 1.00 for IPRs were considered statistically significant with an IPR > 1.00 suggesting a risk association (Knowles et al. 2010). As an example, the following calculation compares the proportion of SR upper extremity fractures to the proportion of fractures due to other types of unintentional injury:

$$ IPR=\frac{\left(\frac{Number\ of\  SR\  ED\  visits\ with\ an\ upper\ extremity\ fracture\ }{Total\ number\ of\  SR\  ED\  visits}\right)}{\left(\frac{Number\ of\ other\ unintentional\ injury- related\  ED\  visits\ with\ an\ upper\ extremity\ fracture}{Total\ number\ of\ other\ unintentional\ injury- related\  ED\  visits}\right)} $$

There is no comprehensive and systematic data collection method that enumerates exposure to sports and recreational activity in the US. Therefore, the denominator for all rate calculations consisted of the NC resident population of 5–18 year-olds. All rates are presented per 100,000 person-years to ensure comparability across the thousand-fold range of incidence. Age group and sex specific incidence rates were calculated using the National Center for Health Statistics bridged-race mid-year population estimates for NC as the denominator (National Center for Health Statistics 2016). The numerator included visits from non-NC residents as well as NC residents. Out-of-state residents were included in the numerator (injury cases) because comprehensive data for out-of-state ED visits by NC residents were not readily available and excluding NC ED visits by out-of-state residents would introduce a downwards bias in the rates. Incidence rate estimates and 95% confidence intervals (CIs) were generated using a Poisson model. All analyses were performed using SAS software, version 9.4 (SAS Institute, Inc.; Cary, NC).

## Results

### Injury incidence

During the period 2010–2014, 767,075 (27.6%) ED visits were identified as being due to unintentional injury mechanisms, out of a total 2.8 million ED visits among children 5–18 years of age. Of these 767,075 unintentional-injury related ED visits, 213,518 visits (27.8%) were related to SR activities. From 2010 through 2014, there was an annual average number of 42,704 SR NC ED visits or 2374.5 (95% CI, 2364.4–2384.6) ED visits per 100,000 person-years among school-age youth, 5–18 years of age. SR injuries accounted for 27.8% of all ED visits unintentional injury among school-age children.

### Selected characteristics of emergency department visits

Table 1 displays the characteristics of ED visits due to total unintentional injury-related ED visits in comparison to SR ED visits among youth, 5–18 years of age. About two-thirds of all SR ED visits involved boys, a higher proportion than total unintentional injury-related ED visits. In the majority of SR and unintentional injury-related ED visits, the patient was discharged from the ED without admission to the hospital. The most common mode of transport to the ED for both SR and total unintentional injury-related ED visits was “walk-in” to the ED via private or public transportation; however, the proportion was slightly higher for SR ED visits (88.2%) versus total unintentional injury-related ED visits (85.2%). Among SR ED visits, the most commonly cited expected source of payment was Medicaid (42.7%) followed by insurance company (38.5%). The fall and spring seasons contained the highest proportion of ED visits for both SR and total unintentional injury-related ED visits, but SR ED visits exhibited greater seasonal trends, with more pronounced differences between fall/spring and winter/summer seasons. For both SR and unintentional injury-related ED visits, the time of day with the highest proportion of visits was during evening hours of 6:00–11:59 PM.

Table 2 displays the case definition for SR injuries organized by E-code category. The three most common E-code categories observed among school-age children and youth 5–18 years of age was “Sports/athletics played as a group or team” (e.g. American tackle football, basketball, and soccer), “other outdoor recreational activities” (e.g. outdoor activities such as roller-skating/skateboarding and snow/off-road vehicles), and “falls/struck by/against in sports”.

**Table 2:** Sports/Recreation-related ED visits among school-age children: NC, 2010–2014

E-code^a^	Description	Specificity^b,c^	N (%)
E007 (.0–.7, .9)	Sports/athletics played as a group or team	1	72,821 (34.1)
E007.0	American tackle football	1	24,420 (11.4)
E007.6	Basketball	1	23,342 (10.9)
E007.5	Soccer	1	10,108 (4.7)
E007.3	Baseball and softball	1	8840 (4.1)
E007.7	Volleyball	1	1708 (0.8)
E007.4	Lacrosse and field hockey	1	883 (0.4)
E007.1	American touch/flag football	1	636 (0.3)
E007.2	Rugby	1	285 (0.1)
E007.9	Other activities played as a group or team	5	2599 (1.2)
E006(.2, .3, .6, .9), E008 (.0–.2, .4)	Sports/athletics played individually	1	6162 (2.9)
E008.1	Wrestling	1	3572 (1.7)
E008.4	Martial arts	1	525 (0.2)
E008.2	Racquet and hand sports	1	186 (0.1)
E006.2	Golf	1	159 (0.1)
E008.0	Boxing	1	156 (0.1)
E006.3	Bowling	1	106 (0.0)
E006.6	Track and field events (excludes running)	1	100 (0.0)
E006.9	Other activities played individually	5	1358 (0.6)
E886.0, E917 (.0, .5)	Fall, or struck by/striking against, in sports	6	23,934 (11.2)
E917.0	Struck by/against in sports, no subsequent fall	6	19,060 (8.9)
E917.5	Struck by/against in sports with subsequent fall	6	2627 (1.2)
E886.0	Fall on same level from collision, pushing, or shoving, by or with other person in sports	6	2247 (1.1)
E008.9	Other specified sports/athletic activity (NEC)	5	779 (0.4)
E005 (.0, .2, .4, .9)	Dancing and other rhythmic movement	1	5517 (2.6)
E005.4	Cheerleading	1	2080 (1.0)
E005.2	Gymnastics	1	1796 (0.8)
E005.0, E005.9	Dancing and other activity involving rhythmic movement	1	1641 (0.8)
E005.1, E001 (.0–.1), E009 (.0–.9), E010 (.0–.9)	Cardiorespiratory and muscle strengthening activities, not elsewhere specified	5	15,413 (7.2)
E001.1	Running	5	9130 (4.3)
E001.0	Walking, hiking, and marching	5	4311 (2.0)
E009 (.0–.9)	Other cardiorespiratory exercise	2	321 (0.2)
E010 (.0–.9)	Other muscle strengthening exercises	2	1641 (0.8)
E005.1	Yoga	1	10 (0.0)
E005.3, E006.5, E007.8, E008.3, E884.0	Activities involving play and other activities usually unstructured	5	18,209 (8.5)
E884.0	Fall from playground equipment	5	11,811 (5.5)
E005.3	Trampoline	1	4623 (2.2)
E008.3	Frisbee	1	152 (0.1)
E006.5	Jumping rope	1	147 (0.1)
E007.8	Physical games generally associated with school recess, summer camp and children	5	1476 (0.7)
E006.4, E800-E807 (.3), E810-E819 (.6), E820-E825 (.6), E826 (.1,.9), E827-E829 (.1)	Pedal cycle	3	20,984 (9.8)
E800-E807 (.3), E820-E825 (.6), E826 (.1,.9), E827-E829 (.1)	Nontraffic-related (i.e. off-road)	3	18,591 (8.7)
E810-E819 (.6)	Traffic-related (i.e. on-road)	3	1006 (0.5)
E006.4	Bike riding, unspecified	5	1387 (0.6)
E002 (.0–.9), E830-E838 (.0, .1, .3, .4, .5, .8, .9), E883.0, E902.2, E910 (.0, .1, .2, .8, .9)	Recreational activities involving bodies of water	4	3889 (1.8)
E002.6, E910.0, E830-E838 (.4)	Waterskiing	2	212 (0.1)
E002 (.0–.5, .7–.9), E830-E838 (.0, .1, .3, .5, .8, .9), E883.0, E902.2, E910 (.1, .2, .8, .9)	Other activities involving water and watercraft	4	3677 (1.7)
E003 (.0–.9), E004 (.0–.9), E006 (.0, .1), E820-E821 (.0, .1, .5, .8, .9), E822-E825 (.5), E826-E829 (.2), E885 (.0–.2), E922 (.4, .5)	Other outdoor recreational activities	4	30,774 (14.4)
E006.0, E885 (.1,.2)	Roller skating and skateboarding	4	12,376 (5.8)
E820-E821 (.0, .1, .8, .9)	Snow and other off-road vehicles	3	6477 (3.0)
E003 (.0–.9), E885 (.3, .4)	Snow skiing, snowboarding, and other activities involving snow and ice	3	3774 (1.8)
E006.1, E820-E825 (.5), E826-E829 (.2)	Animal being ridden	3	2515 (1.2)
E885.0	Fall from non-motorized scooter	3	2485 (1.2)
E922 (.4, .5)	Air gun	2	1593 (0.7)
E004 (.0–.9)	Climbing, rappelling and jumping off	3	1554 (0.7)
E849.4	Injury occurred at a place of recreation or sport, no further detail	7	15,036 (7.0)
Total			213,518 (100.0)

*Abbreviations*: *ED* emergency department, *NEC* not elsewhere classified

^a^Activity E-codes (E-codes E001-E030) describing activities resulting in injury were added to ICD-9-CM starting October 1, 2009 (Bronnert 2009)

^b^During the period 2010–2014, NC DETECT collected up to five E-codes describing the type of injury. For emergency department visits with more than one E-code, preference was given to ED visits with more specific E-codes (“1”) over visits with less specific E-codes (“7”). For ED visits with more than one E-code of the same specificity level, assignment was based on the first-listed E-code

^c^For each category header, the level of specificity is the mode for that category

### Location and nature of injury

Table 3 compares SR ED visits and ED visits due to other unintentional injury mechanisms, as classified by the Barell Injury Diagnosis Matrix. Among SR ED visits, the most common location of injury was upper extremities, lower extremities, and head/face/neck. Fractures of the upper extremity were particularly common in comparison to the proportion of injuries that were due to upper extremity fractures among ED visits due to other mechanisms of unintentional injury (6.1%; IPR: 2.44 [95% CI, 2.40–2.48). In addition, both lower extremity, lower extremity strains/sprains, and upper extremity sprains/strains were nearly twice as common among SR ED visits relative to other unintentional injury mechanisms.

**Table 3:** Unintentional injury-related ED visits among school-age children, by Barell injury diagnosis category: NC, 2010–2014

Barell injury diagnosis category^a^	ED visits due to sports and recreation-related injuries (*N* = 189,679)	Other unintentional injury-related ED visits (*N* = 470,751)	Total unintentional injury-related ED visits (*N* = 660,430)	IPR (95% CI)^e^
Upper extremity, No. (%)	69,581 (36.7)	135,251 (28.7)	204,832 (31.0)	1.28 (1.27–1.29)
Fracture	28,024 (14.8)	28,542 (6.1)	56,566 (8.6)	2.44 (2.40–2.48)
Open wound	2142 (1.1)	29,464 (6.3)	31,606 (4.8)	0.18 (0.17–0.19)
Sprain/strain	14,367 (7.6)	20,715 (4.4)	35,082 (5.3)	1.72 (1.69–1.76)
Superficial wounds and contusions	12,050 (6.4)	33,487 (7.1)	45,537 (6.9)	0.89 (0.88–0.91)
Other and unspecified injuries^b^	12,998 (6.9)	23,043 (4.9)	36,041 (5.5)	1.40 (1.37–1.43)
Lower extremity, No. (%)	53,450 (28.2)	116,833 (24.8)	170,283 (25.8)	1.14 (1.13–1.15)
Fracture	7188 (3.8)	10,032 (2.1)	17,220 (2.6)	1.78 (1.73–1.83)
Open wound	3958 (2.1)	23,450 (5.0)	27,408 (4.2)	0.42 (0.41–0.43)
Sprain/strain	23,868 (12.6)	37,266 (7.9)	61,134 (9.3)	1.59 (1.57–1.61)
Superficial wounds and contusions	9268 (4.9)	29,120 (6.2)	38,388 (5.8)	0.79 (0.77–0.81)
Other and unspecified injuries^b^	9168 (4.8)	16,965 (3.6)	26,133 (4.0)	1.34 (1.31–1.37)
TBIs and other head/face/neck, No. (%)	46,858 (24.7)	103,632 (22.0)	150,490 (22.8)	1.12 (1.11–1.13)
TBI	9424 (5.0)	8543 (1.8)	17,967 (2.7)	2.74 (2.66–2.82)
Fracture	1778 (0.9)	1519 (0.3)	3297 (0.5)	2.90 (2.71–3.11)
Open wound	12,732 (6.7)	38,912 (8.3)	51,644 (7.8)	0.81 (0.80–0.83)
Sprain/strain	37 (0.0)	78 (0.0)	115 (0.0)	1.18 (0.80–1.74)
Superficial wounds and contusions	9472 (5.0)	29,119 (6.2)	38,591 (5.8)	0.81 (0.79–0.83)
Other and unspecified injuries^b^	13,415 (7.1)	25,461 (5.4)	38,876 (5.9)	1.31 (1.28–1.33)
Torso, No. (%)	9431 (5.0)	27,366 (5.8)	36,797 (5.6)	0.86 (0.84–0.88)
Fracture	367 (0.2)	552 (0.1)	919 (0.1)	1.65 (1.45–1.88)
Open wound	488 (0.3)	2200 (0.5)	2688 (0.4)	0.55 (0.50–0.61)
Sprain/strain	842 (0.4)	5209 (1.1)	6051 (0.9)	0.40 (0.37–0.43)
Superficial wounds and contusions	5735 (3.0)	14,461 (3.1)	20,196 (3.1)	0.98 (0.96–1.01)
Other and unspecified injuries^b^	1999 (1.1)	4944 (1.1)	6943 (1.1)	1.00 (0.95–1.06)
Vertebral column, No. (%)	4609 (2.4)	26,302 (5.6)	30,911 (4.7)	0.43 (0.42–0.45)
Fracture	269 (0.1)	669 (0.1)	938 (0.1)	1.00 (0.87–1.15)
Open wound	0 (0.0)	0 (0.0)	0 (0.0)	–
Sprain/strain	4331 (2.3)	25,615 (5.4)	29,946 (4.5)	0.42 (0.41–0.43)
Superficial wounds and contusions	0 (0.0)	0 (0.0)	0 (0.0)	–
Other and unspecified injuries^b,c^	< 10	18 (0.0)	27 (0.0)	–
System-wide and late effects of injury, No. (%)	1324 (0.7)	38,897 (8.3)	40,221 (6.1)	0.08 (0.08–0.09)
Other and unspecified location of injury^d^, No. (%)	4426 (2.3)	22,470 (4.8)	26,896 (4.1)	0.49 (0.47–0.50)

*Abbreviations*: *ED* emergency department, *no.* number, *IPR* injury proportion ratio, *CI* confidence interval, *TBI* traumatic brain injury

Missing: 23839 visits due to sports and recreation-related injuries and 106,645 ED visits due to other unintentional injury mechanisms were missing a valid injury diagnosis code

^a^Categorization based on first-listed injury diagnosis code

^b^Other and unspecified injuries include injuries to the internal organs, nerves, and blood vessels, as well as burns, amputations, dislocations, crushing injuries, and unspecified injuries

^c^In order to protect patient anonymity, cells with counts of 1–9 ED visits are suppressed

^d^Other and unspecified location consists of spinal cord injuries, system wide injuries, late effects of injuries, and other and unspecified injuries

^e^Injury Proportion Ratio and 95% CI compares percent in sports/recreational to percent in non-sport /rec recreational (Knowles et al. 2010)

NC DETECT contains data fields for up to eleven diagnoses. Among SR ED visits, 5.0% of visits had a diagnosis of TBI in the first data field and 12.0% of SR ED visits had a diagnosis of TBI in any one of the eleven available data fields. The proportion of SR ED visits with a diagnosis of TBI was higher than that for other unintentional injury mechanisms (1.8%; IPR = 2.74 [95% CI, 2.66–2.82]). Although not as common, fractures to the head/face/neck were also higher among SR ED visits (0.9%; IPR = 2.90 [95% CI, 2.71–3.11]).

### Activity at time of injury

Tables 4 and 5 display the absolute numbers and incidence rates of injury-related ED visits as well as the proportion of these visits with a diagnosis of TBI, stratified by the type of sport or recreational activity for age group and sex. Among 5–9 year-olds, the most common category of sport and recreational injury was activities involving “play and other activities, usually unstructured” such as “falls from playground equipment”. On the other-hand, “sports/athletics played as a group or team” were by far the most common activity for 10–14 and 15–18 year-olds. Among 10–14-year-olds, American tackle football was the most common cause of injury related to team sports; however, among 15–18 year-olds, basketball was the most common cause of injury related to team sports. For recreational activities, the most common cause of injury was pedal cycling for both 5–9 and 10–14-year-olds. Among 15–18-year-olds, the most common cause of injury was “falls and other injuries resulting from roller skating and skateboarding”. Regarding sports/athletic categories with a diagnosis of TBI, the category of sport with the highest proportion of TBI was American touch/flag football among 5–9-year-olds and rugby among 10–14 and 15–18 year-olds (33.3 and 29.1% respectively). Among recreational activities, the activity with the highest proportion of TBI across all age groups was water-skiing (Table 4).

**Table 4:** Age-group specific rates of sports/recreation-related injuries and proportions of TBI among school-age children: NC, 2010–2014

Sport/ recreational activity^a,b^	Age group											
	5–9 years of age				10–14 years of age				15–18 years of age			
	No.	Rate^c^	% with TBI	*P*-value^d^	No.	Rate^c^	% with TBI	*P*-value^d^	No.	Rate^c^	% with TBI	*P*-value^d^
Team sports	6551	204.6	13.2	<.001	35,750	1108.3	11.5	0.002	30,520	1190.2	13.7	0.87
American tackle football	2274	71	12.8	0.05	12,953	401.6	13.4	<.001	9193	358.5	16.9	<.001
Basketball	1427	44.6	12.5	0.21	10,204	316.3	8.6	<.001	11,711	456.7	8.5	<.001
Soccer	1196	37.3	9.8	0.06	4897	151.8	12.2	0.011	4015	156.6	19.0	<.001
Baseball/softball	1266	39.5	17.9	<.001	4821	149.5	11.8	0.14	2753	107.4	14.1	0.44
Volleyball	36	1.1	11.1	>.99	879	27.3	7.5	<.001	793	30.9	10.8	0.02
Lacrosse/field hockey	28	0.9	10.7	>.99	302	9.4	21.5	<.001	553	21.6	25.1	<.001
Touch/flag football	85	2.7	21.2	0.005	309	9.6	14.2	0.08	242	9.4	17.4	0.09
Rugby	0	–	0.0	–	48	1.5	33.3	<.001	237	9.2	29.1	<.001
Other team sports	239	7.5	12.6	0.61	1337	41.4	10.8	0.76	1023	39.9	13.3	0.75
Individual sports	755	23.6	8.7	0.02	2542	78.8	10.2	0.15	2865	111.7	13	0.32
Wrestling	277	8.7	9.4	0.27	1419	44	11.5	0.64	1876	73.2	13.7	0.94
Martial arts	106	3.3	8.5	0.33	259	8.0	10	0.58	160	6.2	13.1	0.85
Racquet	16	0.5	6.3	>.99	79	2.4	8.9	0.53	91	3.5	7.7	0.10
Golf	58	1.8	17.2	0.17	58	1.8	15.5	0.28	43	1.7	16.3	0.61
Boxing^e^	<10				40	1.2	2.5	0.12	115	4.5	7.8	0.07
Bowling	44	1.4	2.3	0.06	34	1.1	0	0.03	28	1.1	0.0	0.03
Track and field^e^	<10				39	1.2	5.1	0.31	58	2.3	10.3	0.46
Other individual sports	250	7.8	7.6	0.05	614	19	8.5	0.04	494	19.3	13.4	0.86
Fall/struck by/against in sports	2724	85.1	15.2	<.001	11,714	363.2	15.8	<.001	9496	370.3	19.7	<.001
Other team/individual sports, NEC	128	4.0	9.4	0.45	375	11.6	10.1	0.55	276	10.8	12	0.41
Dancing and rhythmic movement	1009	31.5	5.9	<.001	2604	80.7	10.1	0.09	1904	74.2	14.5	0.24
Cheerleading	103	3.2	10.7	0.80	1006	31.2	16.6	<.001	971	37.9	20.2	<.001
Gymnastics	563	17.6	5.0	<.001	938	29.1	7.0	<.001	295	11.5	10.2	0.08
Other dancing/rhythmic movement	343	10.7	6.1	0.002	660	20.5	4.4	<.001	638	24.9	8.0	<.001
Cardio and strength training	4453	139.1	13.0	0.001	6084	188.6	6.3	<.001	4876	190.1	4.8	<.001
Running	3253	101.6	14.5	<.001	3948	122.4	6.7	<.001	1929	75.2	5.4	<.001
Walking	1071	33.4	8.9	0.007	1631	50.6	6.4	<.001	1609	62.7	5.3	<.001
Other cardio	64	2.0	9.4	0.60	134	4.2	2.2	0.001	123	4.8	4.1	0.002
Strength training	63	2.0	12.7	0.76	367	11.4	4.1	<.001	1211	47.2	3.3	<.001
Yoga^e^	<10				<10				<10			
Play/unstructured activities	12,188	380.6	8.1	<.001	5039	156.2	7.5	<.001	982	38.3	9.0	<.001
Fall from playground equipment	9290	290.1	8.9	<.001	2246	69.6	9.7	0.03	275	10.7	13.8	0.93
Trampoline	2396	74.8	4.6	<.001	1816	56.3	3.6	<.001	411	16.0	4.1	<.001
Frisbee	12	0.4	8.3	>.99	61	1.9	11.5	0.93	79	3.1	12.7	0.80
Jumping rope	58	1.8	8.6	0.49	74	2.3	4.1	0.05	15	0.6	6.7	0.71
Activities involving physical games	432	13.5	9.5	0.19	842	26.1	10.0	0.30	202	7.9	10.9	0.25
Pedal cycle	9145	285.6	12.8	<.001	8713	270.1	10.6	0.14	3126	121.9	14.3	0.25
Nontraffic	8297	259.1	12.7	<.001	7775	241	10.4	0.045	2519	98.2	14.1	0.46
Traffic	210	6.6	25.7	<.001	382	11.8	24.1	<.001	414	16.1	17.6	0.02
Unspecified	638	19.9	8.9	0.04	556	17.2	4.3	<.001	193	7.5	9.8	0.12
Activities involving water	1001	31.3	13.1	0.11	1528	47.4	14.8	<.001	1360	53	15.0	0.14
Waterskiing	17	0.5	47.1	<.001	77	2.4	27.3	<.001	118	4.6	23.7	0.001
Other activities involving water	984	30.7	12.5	0.32	1451	45.0	14.1	<.001	1242	48.4	14.2	0.58
Other recreational activities	7244	226.2	11.7	0.56	14,149	438.6	10.5	0.02	9381	365.8	13.9	0.45
Roller skating/skateboarding	2428	75.8	7.5	<.001	6514	201.9	8.0	<.001	3434	133.9	11.5	<.001
Snow/off-road vehicles	1257	39.3	16.8	<.001	2791	86.5	14.5	<.001	2429	94.7	15.9	0.001
Snow skiing, snowboarding, etc.	541	16.9	22.4	<.001	1706	52.9	14.0	<.001	1527	59.5	17.3	<.001
Animal being ridden	414	12.9	21.5	<.001	978	30.4	20.9	<.001	1123	43.8	20.7	<.001
Scooter	1433	45.1	11.4	0.96	929	28.8	9.3	0.07	123	4.8	2.4	<.001
Air gun	377	11.8	2.7	<.001	768	23.8	2.3	<.001	448	17.5	1.1	<.001
Climbing/rappelling	794	24.8	8.7	0.01	463	14.4	4.8	<.001	297	11.6	5.7	<.001
Place of recreation/sport	4293	134.1	13.0	0.001	6387	198	9.4	<.001	4356	169.9	9.9	<.001
Total	49,491	1545.5	11.5	–	94,885	2941.6	11.1	–	69,142	2696.3	13.6	–

^a^Among ED visits with more than one sports and recreation-related E-code, categorization was based on the most specific E-code. In instances when two or more E-codes were the same level of specificity, categorization was based on the first-listed E-code

^b^Sport/recreational activity designations have been abbreviated for display; for complete descriptions, please see Table 1

^C^Population-based incidence rates are per 100,000 person-years

^d^Pearson chi-square tests (expected cell counts > 5) and Fisher’s Exact tests used (expected cell counts < 5) were used for calculation of *p*-values

^e^In order to protect patient anonymity, cells with counts of 1–9 ED visits are suppressed

**Table 5:** Sex-specific rates of sports/recreation-related injuries and proportions of TBI among school-age children: NC, 2010–2014

Injury sport/recreational activity^a,b^	Sex							
	Boys				Girls			
	No.	Rate (95% CI)^c^	% with TBI	*P*-value^d^	No.	Rate (95% CI)^c^	% with TBI	*P*-value^d^
Team sports	56,562	1231.9 (1221.8–1242.1)	12.5	0.32	16,256	369.4 (363.8–375.1)	12.9	<.001
American tackle football	23,603	514.1 (507.5–520.7)	14.9	<.001	817	18.6 (17.3–19.9)	6.9	<.001
Basketball	18,204	396.5 (390.8–402.3)	7.7	<.001	5137	116.7 (113.6–120.0)	12.7	<.001
Soccer	5930	129.2 (125.9–132.5)	13.6	0.02	4177	94.9 (92.1–97.8)	16.1	<.001
Baseball/softball	5315	115.8 (112.7–118.9)	14.9	<.001	3524	80.1 (77.5–82.8)	11.1	0.52
Volleyball	288	6.3 (5.6–7.0)	6.9	0.004	1420	32.3 (30.6–34.0)	9.6	0.14
Lacrosse/field hockey	695	15.1 (14.1–16.3)	22.6	<.001	188	4.3 (3.7–4.9)	26.6	<.001
Touch/flag football	519	11.3 (10.4–12.3)	17.0	0.003	117	2.7 (2.2–3.2)	13.7	0.31
Rugby	232	5.1 (4.4–5.7)	29.3	<.001	53	1.2 (0.9–1.6)	32.1	<.001
Other team sports	1776	38.7 (36.9–40.5)	11.9	0.36	823	18.7 (17.5–20.0)	12.2	0.21
Individual sports	5023	109.4 (106.4–112.5)	12.0	0.17	1139	25.9 (24.4–27.4)	8.6	0.02
Wrestling	3256	70.9 (68.5–73.4)	12.9	0.55	316	7.2 (6.4–8.0)	7.9	0.10
Martial arts	361	7.9 (7.1–8.7)	12.5	0.94	164	3.7 (3.2–4.3)	6.7	0.09
Racquet	90	2.0 (1.6–2.4)	10.0	0.46	96	2.2 (1.8–2.7)	6.3	0.15
Golf	111	2.4 (2.0–2.9)	15.3	0.39	48	1.1 (0.8–1.4)	18.8	0.08
Boxing	144	3.1 (2.7–3.7)	6.3	0.02	12	0.3 (0.2–0.5)	8.3	>.99
Bowling	52	1.1 (0.9–1.5)	1.9	0.02	54	1.2 (0.9–1.6)	0.0	0.004
Track and field	59	1.3 (1.0–1.7)	6.8	0.24	41	0.9 (0.7–1.3)	9.8	>.99
Other individual sports	950	20.7 (19.4–22.0)	10.0	0.02	408	9.3 (8.4–10.2)	10.3	0.74
Fall/struck by/against in sports	18,414	401.0 (395.3–406.9)	17.2	<.001	5518	125.4 (122.1–128.7)	17.7	<.001
Other team/individual sports, NEC	496	10.8 (9.9–11.8)	10.7	0.20	283	6.4 (5.7–7.2)	10.6	0.92
Dancing and rhythmic movement	587	12.8 (11.8–13.9)	8.9	0.006	4930	112.0 (108.9–115.2)	11.1	0.48
Cheerleading	38	0.8 (0.6–1.1)	10.5	>.99	2042	46.4 (44.4–48.5)	18.1	<.001
Gymnastics	250	5.4 (4.8–6.2)	10.4	0.30	1546	35.1 (33.4–36.9)	6.3	<.001
Other dancing/rhythmic movement	299	6.5 (5.8–7.3)	7.4	0.006	1342	30.5 (28.9–32.2)	5.9	<.001
Cardio and strength training	8601	187.3 (183.4–191.3)	9.5	<.001	6810	154.7 (151.1–158.5)	5.7	<.001
Running	5257	114.5 (111.4–117.6)	11.4	0.009	3872	88.0 (85.3–90.8)	6.2	<.001
Walking	1930	42.0 (40.2–44.0)	8.4	<.001	2381	54.1 (52.0–56.3)	5.2	<.001
Other cardio	152	3.3 (2.8–3.9)	6.6	0.03	168	3.8 (3.3–4.4)	2.4	<.001
Strength training	1258	27.4 (25.9–29.0)	3.4	<.001	383	8.7 (7.9–9.6)	5.2	<.001
Yoga^e^	<10				<10			
Play/unstructured activities	9633	209.8 (205.7–214.0)	8.8	<.001	8575	194.9 (190.8–199.0)	7.0	<.001
Fall from playground equipment	6150	133.9 (130.6–137.3)	9.9	<.001	5660	128.6 (125.3–132.0)	8.4	
Trampoline	2420	52.7 (50.6–54.8)	5.4	<.001	2203	50.1 (48.0–52.2)	2.9	<.001
Frisbee	119	2.6 (2.2–3.1)	10.1	0.41	33	0.7 (0.5–1.1)	18.2	0.16
Jumping rope	43	0.9 (0.7–1.3)	11.6	0.85	104	2.4 (2.0–2.9)	3.8	0.02
Activities involving physical games	901	19.6 (18.4–20.9)	10.3	0.04	575	13.1 (12.0–14.2)	9.4	0.28
Pedal cycle	14,561	317.1 (312.0–322.3)	13.4	0.004	6420	145.9 (142.4–149.5)	9.3	<.001
Nontraffic	12,757	277.8 (273.1–282.7)	13.3	0.02	5832	132.5 (129.2–136.0)	9.1	<.001
Traffic	845	18.4 (17.2–19.7)	21.1	<.001	160	3.6 (3.1–4.2)	25.6	<.001
Unspecified	959	20.9 (19.6–22.3)	7.8	<.001	428	9.7 (8.8–10.7)	5.8	<.001
Activities involving water	2397	52.2 (50.2–54.3)	14.4	0.006	1492	33.9 (32.2–35.7)	14.4	<.001
Waterskiing	133	2.9 (2.4–3.4)	26.3	<.001	79	1.8 (1.4–2.2)	27.8	<.001
Other activities involving water	2264	49.3 (47.3–51.4)	13.7	0.10	1413	32.1 (30.5–33.8)	13.7	<.001
Other recreational activities	19,297	420.3 (414.4–426.3)	12.3	0.15	11,475	260.8 (256.0–265.6)	11.1	0.27
Roller skating/ skateboarding	8012	174.5 (170.7–178.4)	11.0	<.001	4364	99.2 (96.3–102.2)	4.9	<.001
Snow/off-road vehicles	4541	98.9 (96.1–101.8)	15.1	<.001	1935	44.0 (42.1–46.0)	16.4	<.001
Snow skiing, snowboarding, etc.	2470	53.8 (51.7–56.0)	17.7	<.001	1304	29.6 (28.1–31.3)	14.4	<.001
Animal being ridden	627	13.7 (12.6–14.8)	16.9	0.001	1887	42.9 (41.0–44.9)	22.2	<.001
Scooter	1321	28.8 (27.3–30.4)	12.5	0.91	1164	26.5 (25.0–28.0)	7.6	<.001
Air gun	1380	30.1 (28.5–31.7)	2.2	<.001	213	4.8 (4.2–5.5)	1.4	<.001
Climbing/rappelling	946	20.6 (19.3–22.0)	7.0	<.001	608	13.8 (12.8–15.0)	6.9	0.002
Place of recreation/sport	9434	205.5 (201.4–209.7)	10.7	<.001	5602	127.3 (124.0–130.7)	10.3	0.23
Total	145,005	3158.2 (3141.9–3174.5)	12.6	–	68,500	1556.6 (1545.0–1568.3)	10.8	–

Missing: 13 missing sex

*Abbreviations*: *No.* number, *CI* confidence interval, *TBI* traumatic brain injury, *NEC* not elsewhere classifiable

^a^Among ED visits with more than one sports and recreation-related E-code, categorization was based on the most specific E-code. In instances when two or more E-codes were the same level of specificity, categorization was based on the first-listed E-code

^b^Sport/recreational activity designations have been abbreviated for display; for complete descriptions, please see Table 1

^c^Population-based incidence rates are per 100,000 person-years

^d^Pearson chi-square tests (expected cell counts > 5) and Fisher’s Exact tests used (expected cell counts < 5) were used for calculation of *p*-values

^e^In order to protect patient anonymity, cells with counts of 1–9 ED visits are suppressed

There was also a difference in injury patterns by sex. Except for volleyball, rates of “sports/athletics played as a group or team” were higher among boys for all listed sports categories. Among boys, rates of ED visits due to American tackle football were highest, while for girls, rates of basketball-related ED visits were highest for team sports/athletics. Not all categories of sports/recreational activities were higher for boys, however. Girls were nearly nine times more likely to visit an ED due to “activities involving dancing and rhythmic movement” than boys (Table 5). In terms of TBI diagnosis, the three sports/athletics activities with the greatest proportion of TBI-related ED visits among boys were rugby (29.3%), lacrosse/field hockey (22.6%), and American touch/flag football (17.0%). Among girls, the three sports/athletics activities with the greatest proportion of TBI-related ED visits were rugby (32.1%), lacrosse/field hockey (26.6%), and soccer (16.1%) (Table 5).

## Discussion

This study used a broad case definition to identify SR ED visits among school age children and youth in a large, well-defined US population. Results indicate that injuries due to sport and recreational activities have substantial high incidence and represent a potentially serious public health problem in the population. There are approximately 43,000 ED visits per year in NC for youth sports injury, 12% of which received a diagnosis of TBI. In addition, much of the literature has focused on more severe SR injuries resulting in hospitalization and death. These injuries represent the “tip of the iceberg” of the total number of SR injuries (Muller et al. 2005). ED visit data provide a more comprehensive picture of the total number, type, and severity of injuries associated with SR activities (Andrew et al. 2012; Dempsey et al. 2005; Gabbe et al. 2005; Gao et al. 2010; Yang et al. 2007).

Consistent with previous population-based studies of SR injuries, fractures and strains/sprains of the upper and lower extremities were the most common types of injuries identified in this study (Bijur et al. 1995; Burt and Overpeck 2001; Conn et al. 2003). There were also more diagnoses of TBI among SR ED visits than other mechanisms of unintentional injury. NC DETECT ED visit data do not capture information on medical cost or length of hospital stay. However, due to the higher proportion of TBI diagnosis reported among SR ED visits, it is possible that these visits may have greater long-term cost than other types of unintentional injuries among children. Previous studies have found that diagnoses of even mild to moderate TBI are associated with high medical costs and may result in sequelae requiring long-term medical care (Leibson et al. 2012; Schneier et al. 2006; Taylor et al. 2002).

Consistent with the literature, population-based rates of SR injuries were higher among boys in comparison to girls (Burt and Overpeck 2001; Conn et al. 2003; Howard et al. 2014). This likely reflects different patterns of participation; that is, the elevated incidence rate among boys is likely a reflection of their greater participation in organized sports activities. These findings may also reflect differences in the perception of risk, variation in the likelihood of injury, and gender differentials in care-seeking by parents (Morrongiello and Rennie 1998; National Federation of High Schools 2016).

Incidence rates of SR injury peaked among children 10–14 years of age. The rate of SR injury declined by 26% among 10–14 and 15–18 year-old girls. Meanwhile, the corresponding decrease among boys was less than 1 %. While physical activity levels tend to decrease in adolescence for both sexes, the baseline level of physical activity and the age at which activity levels start to decline is lower for girls than boys (Caspersen et al. 2000; Sallis 1993).

The team sports with the greatest proportion of ED visits with a diagnosis of TBI were rugby and lacrosse/field hockey. The sport with the highest population-based incidence of TBI was American tackle football. Overall, boys had higher incidence rates of SR TBIs. However, for select sports such as basketball, baseball/softball, rugby, and soccer, the proportion of ED visits with a diagnosis of TBI was higher for girls than boys. This finding is consistent with prior literature indicating that girls may be at a greater risk for several types of sports injuries, including knee injuries and TBIs, than boys (Caine et al. 2008; Darrow et al. 2009; Gessel et al. 2007; Powell and Barber-Foss 2000). In particular, soccer has a relatively high risk of acute injury, especially among girls (Koutures and Gregory 2010). Soccer-related TBIs are most commonly caused by collision with other players, contact with the ground, inadvertent contact with the ball, and intentional contact with the ball (“heading”) (Gessel et al. 2007). While prevention efforts have often focused on instructing children in proper heading technique, or banning heading altogether, it is unclear whether these efforts have made much of an impact on preventing TBIs among children (Comstock et al. 2015; Delaney and Frankovich 2005).

Similar to other states, NC has developed legislation and prevention programs for preventing and managing sports injuries, particularly TBIs (Bloom 2015; Gopfert et al. 2017). In general, these programs have increased the availability of certified athletics trainers at schools; have improved education of coaching staff, student-athletes, and parents; and have led to the development of return-to-play guidelines after TBI. In addition to school-based programs, the medical community has an important role in tackling SR injuries. For example, NC student-athletes with a diagnosis of TBI must be cleared by a physician before returning to play. Therefore, EDs have developed programs linking patients with community services, such as designated concussion clinics, designed to promote TBI recovery and prevent future injuries (WakeMed Health and Hospitals 2009). While these school- and healthcare-based programs are commendable, they often fail to address injury among younger student-athletes and do little to prevent injury due to unorganized sports and recreational activities.

This study has several limitations. NC DETECT ED visit data are collected by hospitals for clinical, billing, and other administrative purposes. The use of these data for public health surveillance is a secondary function. However, data missingness was low (< 15%) for individual data elements used in analyses. Another limitation of this study is related to the use of statewide NC ED visit data. While population-based studies have many strengths, results may not be generalizable to other jurisdictions. Although NC is a large state, (ranked 9th in the US in terms of population) the distribution of physical activity, as well as injury and healthcare usage, may differ from other regions of the US. Ice hockey and lacrosse, for example, have strong regional bases in the mid-east, mid-west, and north-east regions, but currently are less popular in the south, whereas there is less regional variation in participation in sports such as baseball, softball, soccer, football, and basketball.

Finally, the broad definition of sports and recreational activities used in this study included organized school sports, organized community sports, unorganized sports, and recreational outdoor activities. While this comprehensive definition is a strength, it precluded the use of specific activity-time denominators for the calculation of exposure-based rates due to the lack of exposure data sources at the population level in NC (or any other US jurisdiction). While this study identified American tackle football, basketball, and soccer as the three organized sports activities with the highest population-based rates of injury, it is possible that the use of an exposure-based denominator would produce a different results.

## Conclusion

Sports and recreational activities are an important source of morbidity among school-aged children and youth in North Carolina. This is one of the first descriptive epidemiologic studies to use a comprehensive definition to characterize sports and recreation-related injury in a well-defined United States population. In addition, this study indicates that physical activity promotion programs should take into account differences in risk of sports and recreation-related injury by sex and age group.

## Abbreviations

CDC: Centers for Disease Control and Prevention
CI: Confidence interval
E-code: External cause of injury code
ED: Emergency department
ICD-9-CM: International Classification of Diseases, Ninth Revision, Clinical Modification
IPR: Injury proportion ratio
IR: Incidence rate
NC DETECT: North Carolina Disease Event Tracking and Epidemiologic Collection Tool
NC: North Carolina
NEC: Not elsewhere classified
NFHS: National Federation of High Schools
SR: Sports and recreation-related
TBI: Traumatic brain injury
US: United States
